# Screening and Validation of Uric Acid-Lowering LAB from *Trachemys scripta elegans*

**DOI:** 10.3390/microorganisms14102336

**Published:** 2026-10-13

**Authors:** Siyu Liu, Ping Shi, Yuansen Liu, Hafiz Umer Javed, Jiaming Huang, Chunxing Li, Yuhao Liao, Haitao Wang, Binyu Zhai, Rongping Bu

**Affiliations:** 1 Guangxi Key Laboratory of Beibu Gulf Marine Product Healthy Aquaculture and Green Processing, Guangxi Zhuang Autonomous Region Engineering Research Center of Marine Food Nutrition and Processing Technology Innovation, Guangxi College and University Key Laboratory of High-Value Utilization of Seafood and Prepared Food in Beibu Gulf, College of Food Engineering, Beibu Gulf University, Qinzhou 535011, China; 15549684792@163.com (S.L.); bbgu040sp@163.com (P.S.); liuyuansen21@bbgu.edu.cn (Y.L.); qaziumerjaved@bbgu.edu.cn (H.U.J.); 19127702691@163.com (J.H.); 19577048227@163.com (C.L.); 2 Guangxi Key Laboratory of Marine Environmental Disaster Processes and Ecological Protection Technology College of Marine Sciences, Beibu Gulf University, Qinzhou 535011, China; liaoyuhao131@163.com (Y.L.); 19177757917@163.com (H.W.); zby020301@163.com (B.Z.)

**Keywords:** hyperuricemia, uric acid, probiotic, purine, xanthine oxidase, *Caenorhabditis elegans*

## Abstract

Hyperuricemia (HUA), a metabolic disorder characterized by elevated serum uric acid (UA) levels resulting from purine metabolism dysfunction, is closely associated with gout, hypertension, and cardiovascular diseases. Current pharmacological treatments are frequently associated with adverse effects, underscoring the need for safer alternatives. In this study, lactic acid bacteria (LAB) with UA-lowering potential were isolated from the intestine of *Trachemys scripta elegans* and screened through a multi-indicator approach combining UA degradation, purine metabolism, and xanthine oxidase (XOD) inhibition assays. Five candidate strains were identified, among which SL-5 exhibited the most outstanding performance, degrading 76.19% of UA, 96.62% of adenosine, 83.50% of guanosine, and 99.29% of hypoxanthine, while inhibiting XOD activity by 71.51%, demonstrating a dual capacity to both degrade purines and suppress UA synthesis. In vivo validation using a *C. elegans* hyperuricemia model confirmed that SL-5 reduced UA levels by 52.50%. Physiological characterization further revealed robust growth, high self-aggregation capacity (>80%), strong stress tolerance, and potent antioxidant activity. 16S rDNA sequencing identified SL-5 as *Enterococcus hirae.* This study establishes an effective screening framework and provides a novel strain resource for the development of enzyme-based interventions against hyperuricemia.

## 1. Introduction

Hyperuricemia (HUA) is a chronic metabolic disorder characterized by elevated serum uric acid (UA) levels, resulting from disrupted purine metabolism and representing a significant public health concern [1]. Pathologically, HUA induces inflammatory damage, oxidative stress, and gut microbiota dysbiosis, with sustained hyperuricemia further exacerbating UA overproduction [2]. The deposition of urate crystals in synovial joints and periarticular tissues triggers acute inflammatory cascades, which underlie the painful clinical manifestations of gout. Notably, HUA not only serves as the primary etiological factor for gout but also exhibits strong associations with hypertension, diabetes mellitus, and cardiovascular diseases [3]. Current clinical management of HUA primarily relies on pharmacological interventions and dietary modifications. First-line medications include xanthine oxidase (XOD) inhibitors and uricosuric agents for urate lowering, as well as nonsteroidal anti-inflammatory drugs (NSAIDs) for acute gout flares. While clinically effective, these therapeutic regimens are associated with adverse effects such as gastrointestinal bleeding (mainly attributed to NSAIDs), hepatotoxicity, and allergic reactions [4]. Dietary interventions focus on restricting high-purine foods and avoiding fructose and alcoholic beverages. However, long-established dietary habits significantly compromise patient compliance, limiting the practical implementation of nutritional strategies [5]. These therapeutic limitations underscore the urgent need for developing novel, safe, and effective intervention strategies.

In recent years, certain lactic acid bacteria (LAB) strains have demonstrated potential in modulating purine metabolism and reducing serum UA levels through multi-target regulatory mechanisms. For instance, *Limosilactobacillus fermentum* JL-3 alleviates HUA by restoring gut microbiota dysbiosis and enhancing purine catabolism via intestinal colonization [6], while *Lactobacillus rhamnosus* R31 and *L. reuteri* L20M3 modulate UA levels through enrichment of short-chain fatty acid (SCFA)-producing microbiota and suppression of xanthine oxidase activity [7]. Existing studies predominantly evaluate the UA-lowering function of LAB through in vitro screening methods. For instance, *L. brevis* DM9218 was selected based on purine conversion efficiency [8], *L. plantarum* MC14 through nucleoside assimilation and purine nucleotide degradation assessments [9], and *L. fermentum* NCUH003018 was preliminarily identified via its inosine/guanosine degradation capacity and XOD inhibitory activity [10]. However, the anti-hyperuricemic effect of LAB is generally the synergistic outcome of multiple mechanisms, and single-indicator screening methods are limited in their ability to comprehensively evaluate overall UA-lowering capacity. More importantly, not all LAB strains with potent UA-lowering activity are suitable for direct application as probiotics have been reported as opportunistic pathogens, and their safety requires rigorous evaluation [11]. Therefore, for LAB strains exhibiting functional potential—particularly those belonging to genera such as Enterococcus that harbor species recognized as opportunistic pathogens and thus require rigorous safety evaluation—a more prudent and forward-looking strategy is to consider them as donor hosts for mining genes encoding UA-degrading enzymes, such as purine nucleoside phosphorylase (PNP) and urate dehydrogenase (Udh), rather than directly promoting them for probiotic use. This approach not only manages reader expectations regarding the intended application of such strains but also provides a safer avenue for developing enzyme-based interventions against hyperuricemia.

Animal models of HUA serve as crucial tools for screening safe and efficacious UA-lowering agents. Currently established models predominantly utilize rodents such as mice and rats. For instance, *L. brevis* DM9218 was evaluated in murine models for its XOD inhibitory activity [8], while *L. gasseri* PA-3 was investigated in rat experiments to assess intestinal absorption of inosine and related compounds [12]. However, substantial interspecies differences in UA metabolism limit the translational relevance of these models. Most mammals, including rodents, express functional uricase to catabolize UA, whereas humans lack this enzyme due to evolutionary gene loss, resulting in distinct purine metabolic endpoints. The nematode *Caenorhabditis elegans* (*C. elegans*) has emerged as a promising experimental platform with unique advantages. Its genome exhibits remarkable conservation with humans, sharing 60–80% homologous genes, and crucially, it lacks uricase-encoding genes [13], mirroring the terminal products of human purine metabolism and providing a critical theoretical foundation for the construction of HUA models. Currently, the application of the *C. elegans* model in studying purine metabolism regulation by LAB remains largely underexplored.

Beyond model selection, the choice of an appropriate source for isolating UA-metabolizing microorganisms is equally critical. Turtles are vertebrates characterized by relatively long lifespans and remarkable metabolic adaptability. Among them, *Trachemys scripta elegans* (red-eared slider), due to its wide distribution and strong tolerance to environmental fluctuations, has become an important model for studying metabolic adaptation in reptiles. Previous studies have demonstrated its metabolic robustness: Tang et al. [14] reported that lipid and energy metabolism in juvenile *T. scripta elegans* were only minimally affected under low-temperature stress, indicating an ability to maintain relatively stable metabolic states under thermal fluctuations. Similarly, Hong et al. [15] showed that *T. scripta elegans* tolerates temperatures above 35 °C, further highlighting its superior environmental adaptability. Gradela et al. [16] reported that serum UA levels in *T. scripta elegans* typically range from 0.6 to 2.5 mg/dL (approximately 35–150 μmol/L) and exhibit dynamic fluctuations influenced by multiple factors, including feeding status, environmental conditions, and nutritional state [16,17]. These findings suggest that UA in *T. scripta elegans* functions as a finely regulated metabolite rather than a mere indicator of pathological status. These characteristics make *T. scripta elegans* a suitable biological source for investigating UA-related metabolic processes and their associated microorganisms. However, the potential of reptile-derived LAB in UA metabolism regulation remains largely unexplored.

Based on the above background, this study aimed to isolate LAB candidates with UA-lowering potential from the intestinal contents of *T. scripta elegans*. By establishing a research framework that integrates multi-indicator in vitro screening (UA degradation, purine metabolism, XOD inhibition) with in vivo validation using a uricase-deficient *C. elegans* model of hyperuricemia, we systematically evaluated the comprehensive UA-lowering capacity of candidate strains. Functionally, active strains are valuable genetic resources for mining UA-degrading enzyme genes, providing a foundation for the subsequent development of enzyme-based interventions or engineered strains. This study provides experimental evidence for understanding the potential role of the gut microbiota in UA metabolism in reptiles and to open new resource channels for enzyme-based intervention strategies against hyperuricemia.

## 2. Materials and Methods

### 2.1. Materials and Chemical Reagents

*Trachemys scripta elegans* were obtained from the Bird and Flower Market in Qinzhou, Guangxi, China, while MRS broth and agar were sourced from Qingdao Hi-tech Industrial Park Hope Bio-technology Co., Ltd. in Shandong, China. Allopurinol, ABTS, DPPH, and bile salt were purchased from Macklin Biochemical Technology Co., Ltd., Yuanye Biotechnology Co., Ltd., and Sinopharm Chemical Reagent Co., Ltd., all based in Shanghai, China. UA reagent kits were supplied by Nanjing Jiancheng Bioengineering Institute in Jiangsu, China, and chromatographic standards for inosine, guanosine, adenosine, guanine, xanthine, hypoxanthine, and UA were obtained from Shanghai Aladdin Biochemical Technology Co., Ltd. All other reagents were of analytical grade and commercially available.

All LAB strains used in this study were isolated from the intestinal contents of Trachemys scripta elegans. The Escherichia coli OP50 and the wild-type *C. elegans* N2 were obtained from the Caenorhabditis Genetics Center (CGC). *C. elegans* were cultured on Nematode Growth Medium (NGM) agar plates with *E. coli* OP50 as the standard food source.

### 2.2. In Vitro Evaluation of UA Reducing Ability of Strains

#### 2.2.1. Isolation and Purification of Strains with UA-Lowering Ability

*Trachemys scripta elegans* were housed in a recirculating aquaculture system with water temperature maintained at 24–26 °C, equipped with dry basking platforms and a 12 h light, 12 h dark photoperiod to accommodate behavioral thermoregulation. The animals were fed commercial pelleted diets and acclimatized to laboratory conditions for two weeks prior to the experiments, during which their health status was monitored daily. All procedures were performed in strict accordance with the principles of minimizing animal suffering. Under aseptic conditions, the turtles were deeply anesthetized by inhalation of diethyl ether vapor prior to euthanasia. All experimental protocols were reviewed and approved by the Ethics Committee of Beibu Gulf University (Approval No. YXDW097) and complied with Chinese laws and regulations on animal welfare.

Intestinal tissues were aseptically dissected, repeatedly rinsed with sterile physiological saline to remove surface lipids and then sectioned into 3–4 cm segments. Intestinal contents were homogenized in saline to prepare 10% (*w*/*v*) stock suspensions followed by serial dilution. Aliquots of diluted samples were spread on selective agar plates containing 11 mmol/L UA as the sole carbon/nitrogen source. After 36 h of incubation at 37 °C, dominant colonies exhibiting typical morphology with transparent hydrolysis halos were primarily screened. The screened strains were streaked onto MRS broth, cultured at 37 °C for 36 h, and turbid cultures were subsequently subcultured onto MRS agar for secondary screening. Pure cultures demonstrating characteristic LAB morphology were cryopreserved in 30% glycerol suspension.

#### 2.2.2. Determination of the Ability of Strains to Degrade UA

The UA degradation assay was performed according to Wu et al. [6] and Wu et al. [18] with minor modifications. A 0.5 mL suspension was reacted with 2 mM UA in PBS (pH 7.4) at 37 °C, 220 rpm for 12 h. After centrifugation, 8 μL of supernatant was mixed with 12 μL PBS and 150 μL reaction mixture (containing Tris-HCl buffer, peroxidase, and uricase) to form Ae. Absorbance was measured at 510 nm using a microplate reader (Synergy H1, BioTek Instruments, Inc., Winooski, VT, USA). PBS and a 400 μM UA standard were used as the blank (Ab) and 400 μM standard concentration (As, Cb) references, respectively. UA concentration was calculated using Formula (1).

C (μmol/L) = (Ae − Ab )/(As − Ab) × Cb(1)

#### 2.2.3. Degradation of Purine and Purine Nucleoside

The degradation of purines and purine nucleosides was evaluated using a modified method based on Hussain et al. [19]. Cell pellets were resuspended in 2 mL of substrate solution containing 1 mM adenosine, guanosine, inosine, adenine, xanthine, and hypoxanthine. After adjusting the OD_600_ to 1.5, the suspension was incubated at 37 °C for 1 h. The reaction was stopped by centrifugation and the supernatant was treated with 0.1 M HClO_4_, kept on ice for 10 min, and filtered through a 0.22 μm membrane. Filtrates (20 μL) were analyzed by high-performance liquid chromatography (HPLC, Waters e2695, Waters Corporation, Milford, MA, USA) at 243 nm and quantified using an external standard curve.

#### 2.2.4. XOD Inhibition Assay In Vitro

The XOD inhibitory activity was assessed in vitro according to Zhao et al. [10] with slight modifications. A mixture containing 20 μL of supernatant, 212 μL of 0.2 M PBS (pH 7.0), and 8 μL of xanthine substrate (5 mmol/L) was reacted with 10 μL of XOD (0.025 U/mL) at 37 °C for 10 min. Absorbance was measured at 290 nm before (As0, Ab0) and after (As, Ab) the reaction, using PBS as a blank control and allopurinol as the positive control. The inhibition rate was calculated using Formula (2).

Inhibition rate of XOD (%) = [(1 − (As − As0)/(Ab − Ab0)] × 100(2)

### 2.3. In Vivo Verification of the Ability of the Strains to Reduce UA

#### 2.3.1. Cultivation, Passage and Synchronization of *C. elegans*

Following the method outlined by Li et al. [20].

#### 2.3.2. Strain Treatment in a Hyperuricemia *C. elegans* Model

The experimental design comprised seven groups: a control group (CON), a hyperuricemia model group (MOD), and five intervention groups (SL-4, SL-5, SL-7, SL-10, and SL-17), using a method adapted from Gao et al. [21]. The CON group was continually fed *E. coli* OP50. Synchronized eggs from the MOD and intervention groups were initially cultured on NGM plates seeded with OP50 at 20 °C until reaching the L4 larval stage. Approximately 500 L4-stage nematodes from each group were then transferred to fresh NGM plates containing either OP50 (MOD group, 10^9^ CFU/mL) or the respective SL strain (intervention groups, 10^9^ CFU/mL), and incubated at 20 °C for 48 h. Subsequently, the nematodes were moved to six-well plates containing 3.6 mL of S medium and 1.2 mL of xanthine solution (0.25 mg/mL) per well and incubated at 20 °C for 24 h before intracellular UA levels were measured. All experiments were performed in triplicate.

#### 2.3.3. Detection of UA Content in *C. elegans*

The *C. elegans* were transferred to centrifuge tubes and centrifuged. After discarding the supernatant, the pellets were washed twice with M9 buffer to remove residual xanthine and collect the nematodes. Each sample group was subjected to centrifugation again and the supernatant was discarded. The precipitate was retained and washed twice with M9 buffer. Then, 3 mL of 1 mol/L NaOH solution was added, and the samples were thoroughly ground using a handheld homogenizer, followed by vortexing for 3 min. Subsequently, the samples underwent ultrasonic treatment at 200 W and 25 °C for 5 min, after which they were centrifuged again. The filtrate obtained after passing the supernatant through a 0.22 μm membrane was analyzed by HPLC using a 20 μL aliquot. The HPLC system (Waters e2695, Waters Corporation, Milford, MA, USA) was equipped with a UV detector set at 243 nm. Separation was performed on an Intertsil ODS-SP C18 column (5 μm, 4.6 mm × 250 mm) 10 mmol sodium phosphate (pH 4.7) at a ratio of 1:9, with a flow rate of 0.5 mL/min. The column temperature was maintained at 37 °C, and the injection volume was 20 μL [19].

### 2.4. Characterization of Strains

#### 2.4.1. Growth and Acid Production Characteristics

The growth and acid-production profiles of the strains were evaluated according to Xu et al. [22] with minor adjustments. Absorbance at 600 nm was measured at 0, 2, 4, 6, 8, 10, 12 and 14 h. A growth curve was constructed using MRS medium without lactic acid bacteria as the blank. Simultaneously, pH values of the MRS medium were determined to generate the acid production curve.

#### 2.4.2. Self-Aggregation Ability Measurement

The self-aggregation ability of the strain was evaluated following the method described by Xu et al. [22] with appropriate modifications. The bacterial pellets were resuspended in PBS to achieve an absorbance of 0.5 at 600 nm, recorded as A0. After standing for 2 h, 4 h and 6 h, the absorbance was measured at 600 nm, recorded as At1, At2 and At3. The self-aggregation rate of the strain was calculated according to following Formula (3).

Self aggregation rate (%) = [(A0 − At)/A0] × 100(3)

#### 2.4.3. Tolerance to Salt, Nitrite, Acid and Bile Salt

Viability and stress tolerance to salt, nitrite, acid, and bile salt were assessed by referring to our previous work [23] with minor modifications. Salt and nitrite tolerance was determined by inoculating the strain (1%) into MRS broth containing 6 g/100 mL NaCl or 150 mg/L NaNO_2_ and incubating at 37 °C for 24 h. Growth was quantified by measuring OD_600_, with uninoculated MRS broth as the blank. For acid (pH 3.0) and bile salt (0.3%) tolerance assays, 500 μL of activated culture (1.0 × 10^9^ CFU/mL) was added to 1 mL of MRS medium and incubated at 37 °C. Viable counts were determined by the plate counting method before and after 3 h of incubation, recorded as C0h and C3h. The survival rate was calculated using Formula (4).

Survival Rate (%) = [C3h/C0h] × 100(4)

### 2.5. Antioxidant Activity

Fermentation supernatant (FS) and bacterial suspension (BS) were prepared according to Liu et al. [23]. Briefly, the strain was cultured in MRS medium at 37 °C for 24 h and the supernatant was collected after centrifugation as FS. The bacterial pellets were resuspended in PBS and adjusted to an OD_600_ of 1.0 to obtain BS. The antioxidant activities of FS and BS were evaluated by measuring the scavenging rates of ABTS (734 nm), DPPH (517 nm), and •OH (510 nm) radicals using spectrophotometry.

### 2.6. Morphological and Molecular Identification

Gram staining was performed according to the manufacturer’s instructions provided in the Gram staining kit (Beijing Solaibo Science & Technology Co., Ltd., Beijing, China). The experimental phenomena were observed and recorded under the optical microscope (magnification: 1000×), and the positive bacteria were screened out. DNA extraction kit (Jiangsu Cowin Biotech Co., Ltd., Jiangsu, China) was used to extract DNA from the target strains. Polymerase Chain Reaction (PCR) amplification was then performed using common primers 27F and 1492R. The resulting sequences were amplified and then uploaded and compared to the homologous nucleotide sequence of NCBI. BLAST+ (Version 2.17.0) algorithm in the NCBI database was used to identify the isolate, and the phylogenetic tree was constructed using MEGA 11 software.

### 2.7. Statistical Analysis

Each experiment was performed in independent triplicate, and the data were presented as the mean ± standard deviation (SD) of three independent experiments. The data were assumed to follow a normal distribution and were subjected to ANOVA, with mean comparisons performed using the Tukey test in IBM SPSS Statistics (Version 26.0).

## 3. Results

### 3.1. In Vitro Screening of Strains with Anti-Hyperuricemia Ability

#### 3.1.1. Screening of Strains with UA-Degrading Ability

**Figure 1:**
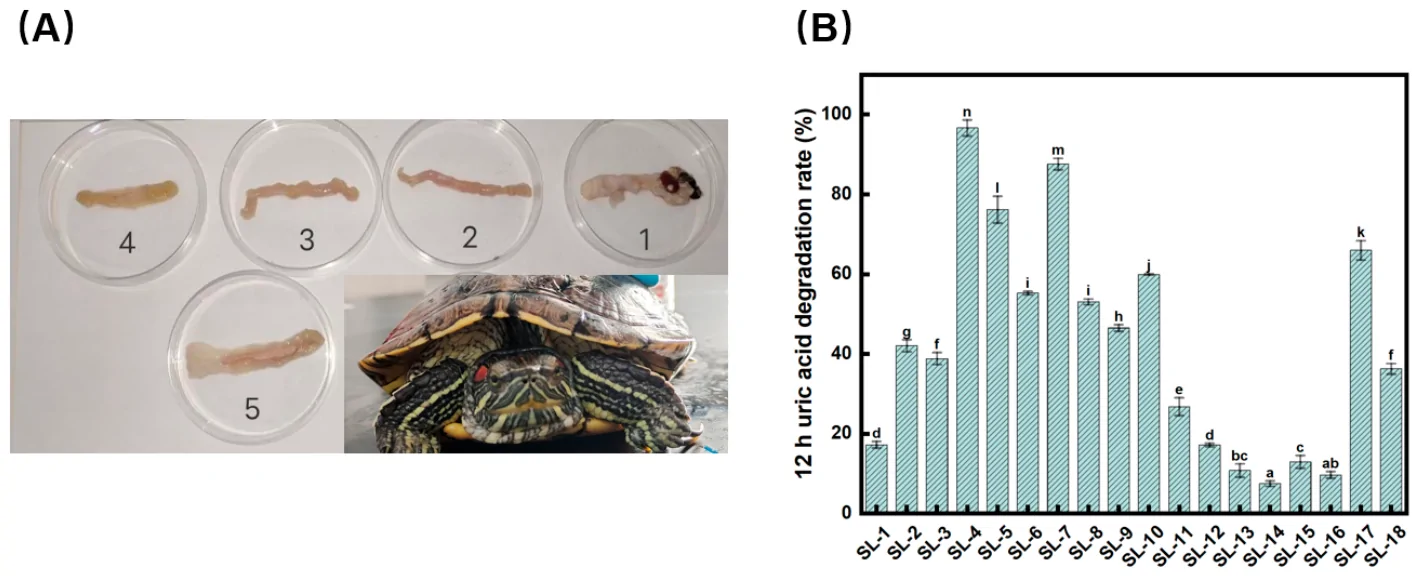
Screening of UA-degrading candidate strains from *Trachemys scripta elegans*. (**A**) Intestinal tract dissection and segmentation procedure. The intestine was divided into five segments: Segment 1, proximal small intestine; Segment 2, middle small intestine; Segment 3, distal small intestine; Segment 4, proximal large intestine; Segment 5, distal large intestine. (**B**) UA degradation rates after 12 h co-culture with candidate strains. Notes: Different lowercase letters above bars indicate statistically significant differences among strains (*p* < 0.05).

In our previous work, we identified the intestine of *T. scripta elegans* as a promising source for isolating UA-reducing strains [23]. Building on this finding, the current study further investigated the UA-degrading potential of these isolates. Healthy *T. scripta elegans* were euthanized under aseptic conditions, and their intestines were divided into five segments (Figure 1A). The contents of each segment were collected for primary screening. After 36 h of culture, several colonies exhibiting typical LAB morphology were observed. Eighteen colonies were selected for further analysis and incubated with sterile PBS containing UA for 12 h using the resting cell method (Figure 1B). The results demonstrated that strains SL-4, SL-5, SL-7, SL-10, and SL-17 exhibited significantly higher UA degradation rates compared to other tested strains (*p* < 0.05), with degradation efficiencies of 96.66 ± 2.03%, 76.19 ± 3.35%, 87.60 ± 1.44%, 59.96 ± 0.18%, and 65.97 ± 2.44%, respectively.

#### 3.1.2. Screening of Strains with Purine-Degrading Ability

The purine-degrading abilities of the five selected strains were evaluated using HPLC. As summarized in Table 1, all five strains exhibited consistently high adenosine degradation rates (93.71–97.07%), with no significant inter-group differences (*p* > 0.05). For guanosine, SL-5 (83.50 ± 1.54%) and SL-7 (83.70 ± 2.35%) demonstrated significantly higher degradation rates compared to the other strains (*p* < 0.05). In the case of inosine, SL-4, SL-5, and SL-7 showed markedly higher degradation rates (36.60 ± 0.09% to 39.67 ± 1.62%) than SL-10 and SL-17 (*p* < 0.05). Adenine degradation varied among strains, with SL-7 achieving the highest rate (42.69 ± 5.77%), followed by SL-4 (36.05 ± 5.56%) and SL-5 (27.83 ± 3.66%) (*p* < 0.05). In contrast, xanthine degradation remained relatively low across all strains, ranging from 3.90 ± 2.16% to 15.89 ± 4.88%. Notably, SL-5 demonstrated exceptional hypoxanthine-degrading activity, achieving a degradation rate of 99.29 ± 0.56%.

#### 3.1.3. Screening of Strains with XOD Inhibition Ability

**Figure 2:**
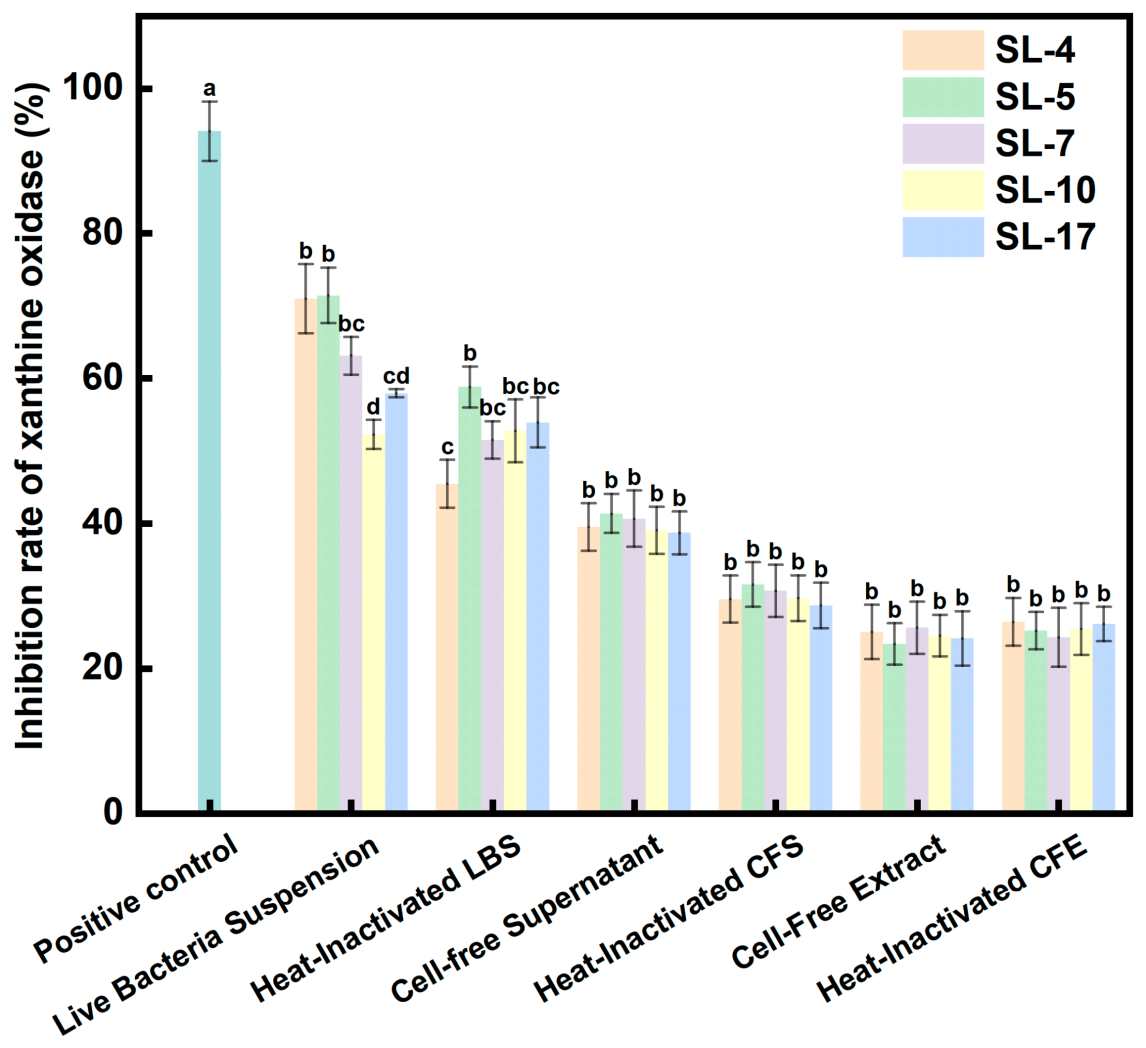
Effect of candidate strains on XOD inhibitory activity. Notes: Different lowercase letters above bars indicate statistically significant differences among strains (*p* < 0.05).

The inhibitory effects of the five strains (SL-4, SL-5, SL-7, SL-10, and SL-17) on XOD activity were comprehensively evaluated using different bacterial fractions, including live and heat-inactivated forms of both extracellular and intracellular components (Figure 2). All strains exhibited varying degrees of XOD inhibitory activity under different treatment conditions, with significant differences observed among strains and fractions. Overall, live bacterial suspensions (LBS) showed the highest inhibitory activity, with inhibition rates ranging from 50.28% to 75.33%, followed by heat-inactivated LBS (42.20% to 61.66%). In contrast, metabolites exhibited moderate inhibitory effects (30.42% to 46.14%), while intracellular components showed relatively lower activity (21.58% to 37.12%). Notably, SL-5 consistently exhibited the strongest inhibitory activity across all tested fractions, indicating strain-dependent differences in XOD inhibition potential. The positive control (allopurinol) showed an inhibition rate of 94.14 ± 4.09%, confirming the validity of the assay system.

### 3.2. Effect of Strains in Hyperuricemic C. elegans

**Figure 3:**
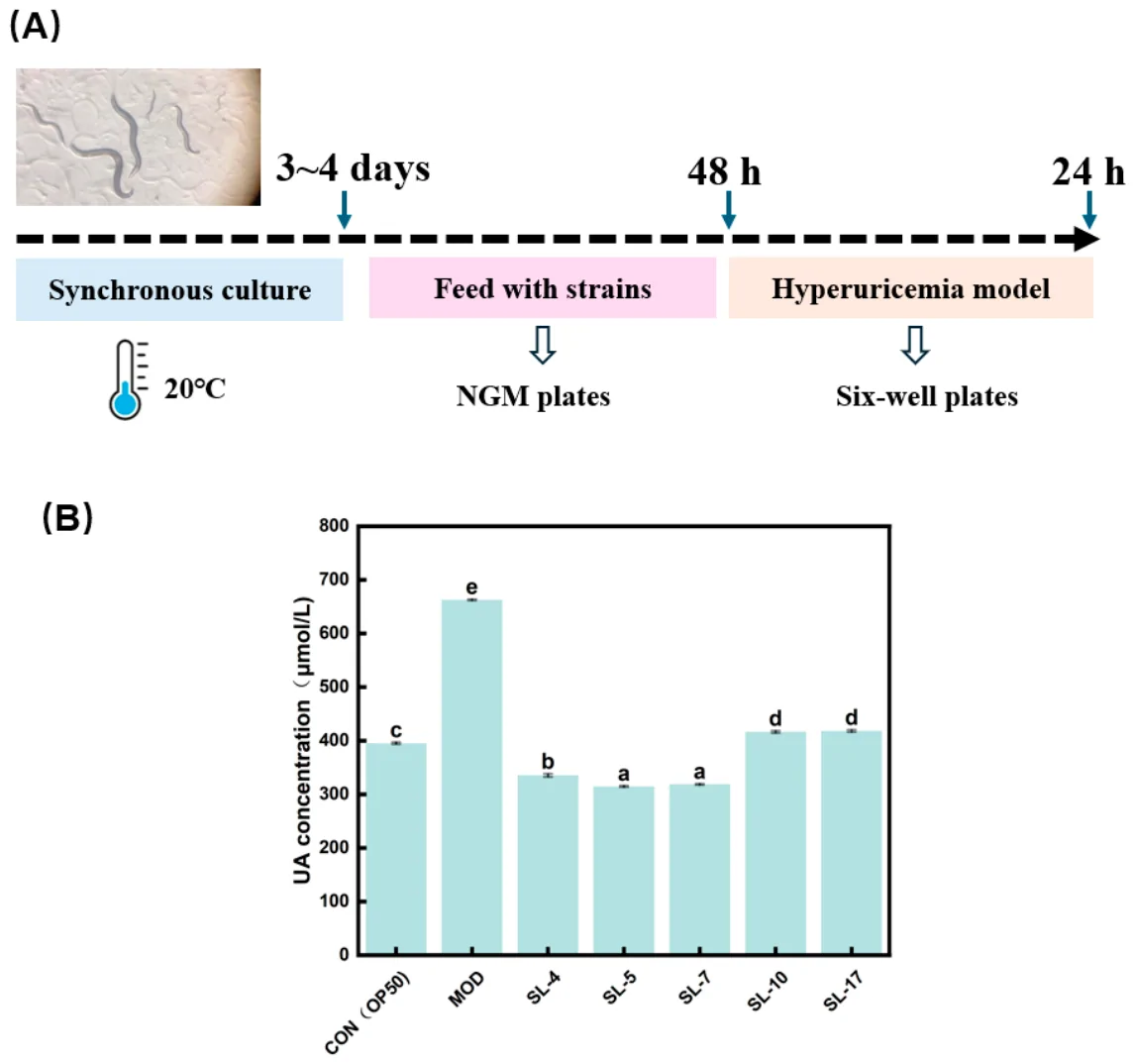
(**A**) Synchronization of *C. elegans*, feeding strains and modeling process of hyperuricemia; (**B**) Effect of Candidate Strains on UA of *C. elegans* Model with High UA. Notes: Different lowercase letters above bars indicate statistically significant differences among strains (*p* < 0.05).

All *C. elegans* were cultured to the L4 stage by feeding with OP50. The nematodes were then fed with different candidate strains (with the CON group receiving *E. coli* OP50) and subsequently exposed to xanthine solution for 24 h (Figure 3A). the UA content in *C. elegans* was then quantified (Figure 3B). The results showed that UA levels in the MOD group significantly increased from 395.56 ± 1.91 μM to 662.93 ± 1.34 μM (*p* < 0.05), confirming the successful establishment of the HUA model. All candidate strains significantly reduced UA levels in hyperuricemic nematodes compared to the MOD group. Among them, SL-4, SL-5, and SL-7 were the most effective, reducing UA concentrations to 335.50 ± 2.92, 314.91 ± 1.48, and 318.69 ± 1.28 μM, respectively. These values were even lower than those in the CON group, indicating a potent hypouricemic effect, particularly for SL-5.

### 3.3. Physiological Properties

#### 3.3.1. Growth Curve, Acid-Producing Ability, and Self-Aggregation

**Figure 4:**
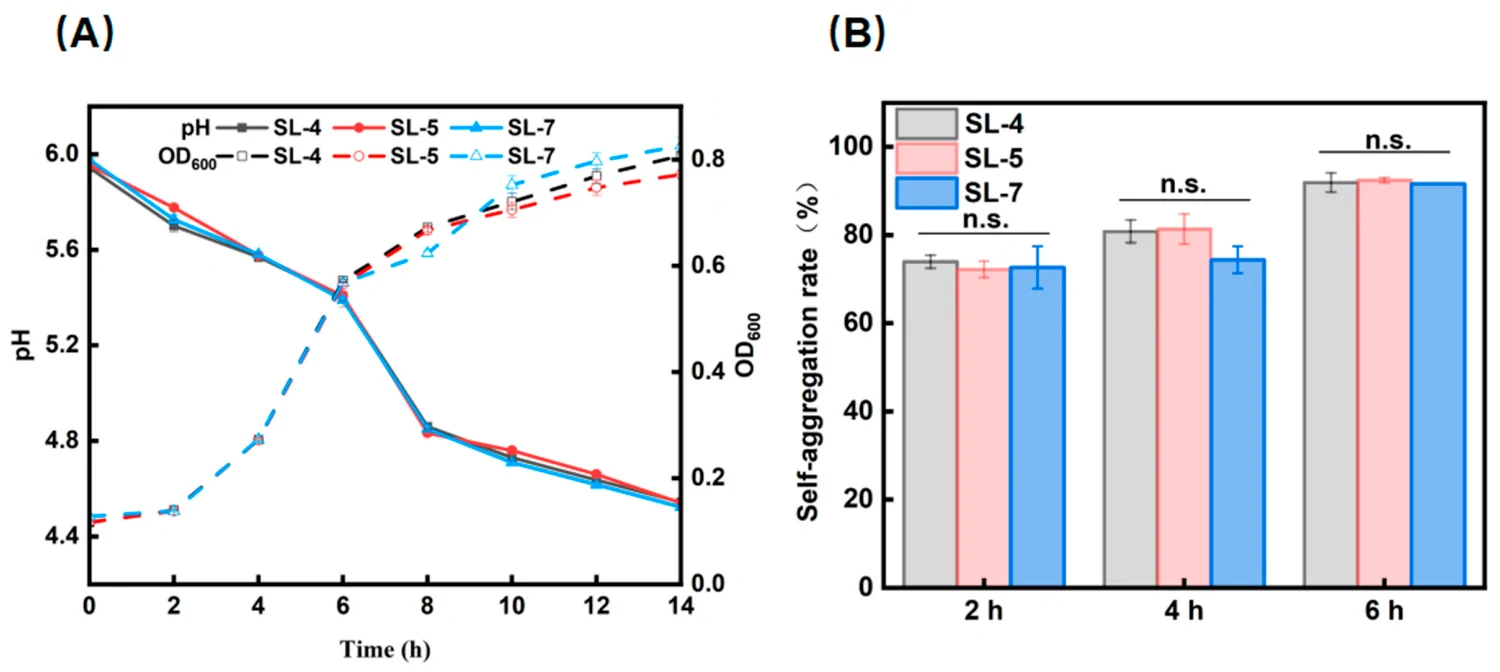
(**A**) Growth curve and acid production characteristics of candidate strains; (**B**) Self-aggregation rate of candidate strains for 2 h, 4 h and 6 h. Notes: n.s. means no significant difference (*p* ≥ 0.05).

The growth patterns of SL-4, SL-5, and SL-7 were monitored through growth curves, and the pH changes in the fermentation broth were recorded simultaneously (Figure 4A). All three strains exhibited rapid growth and strong acid production capacity. As shown in Figure 4B, the self-aggregation rates of the three strains increased with incubation time. After 6 h, the self-aggregation rates of all three strains exceeded 80%.

#### 3.3.2. Salt, Nitrite, Acid and Bile Salt Resistance

**Figure 5:**
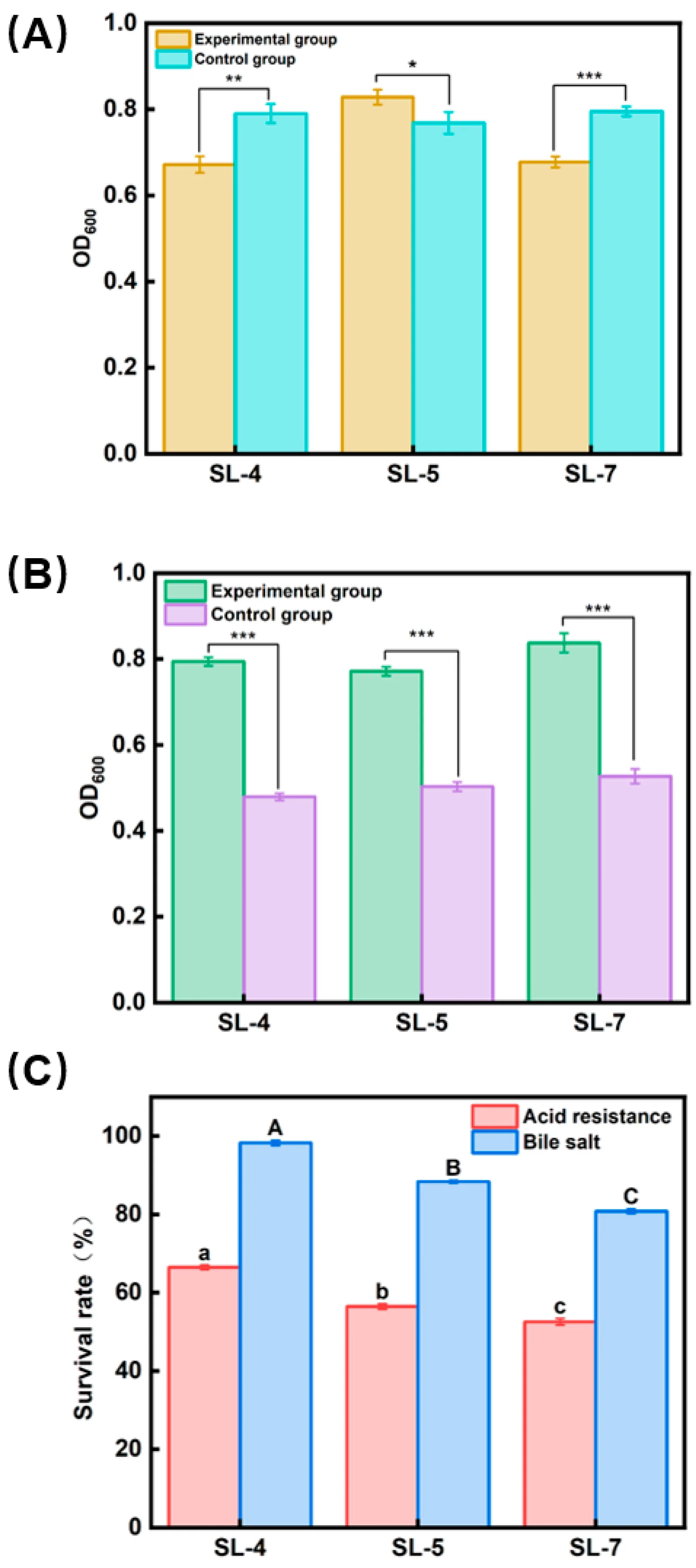
(**A**) Salt tolerance of candidate strains; (**B**) Nitrite resistance; (**C**) Acid resistance and bile salt resistance. Notes: * means significant (*p* < 0.05), ** means very significant (*p* < 0.01), *** means extremely significant (*p* < 0.001) and ns means no significant difference (*p* ≥ 0.05). Different lowercase letters above bars indicate statistically significant differences among strains (*p* < 0.05).

As shown in Figure 5A, SL-5 exhibited significantly enhanced growth under 6 g/100 mL NaCl compared to the control (*p* < 0.05), whereas SL-4 and SL-7 showed slower proliferation. At a concentration of 150 mg/L NaNO2 (Figure 5B), the growth of SL-4, SL-5, and SL-7 all showed significant differences compared to the control (*p* < 0.001). As shown in Figure 5C, at pH 3.0, the survival rates of SL-4, SL-5, and SL-7 were 66.47%, 55.46%, and 52.58%, respectively, with SL-4 exhibiting a significantly higher survival rate than the other strains (*p* < 0.05). At 0.3% bile salt concentration (Figure 5C), the survival rate of SL-4 approached 100%, significantly higher than the other strains, while SL-5 maintained a high tolerance level of 88.37% (*p* < 0.05).

### 3.4. Antioxidant Characteristics

**Figure 6:**
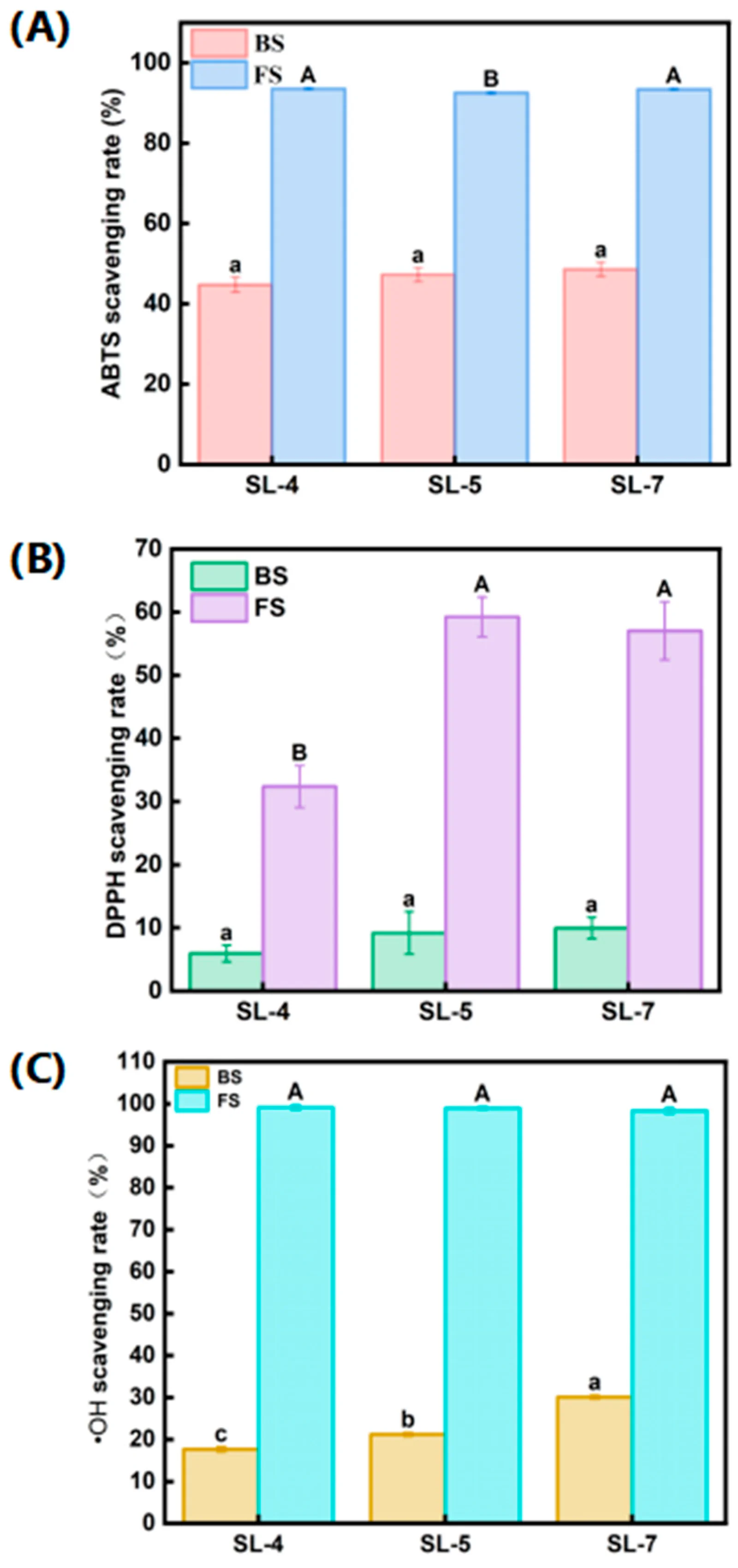
(**A**) Scavenging rate of candidate strains to ABTS cationic free radicals; (**B**) The scavenging rate of DPPH radical; (**C**) The scavenging rate of •OH radical. Notes: Different lowercase and uppercase letters above bars indicate statistically significant differences among strains (*p* < 0.05).

The antioxidant activities of both BS and FS were evaluated by measuring their scavenging rates against ABTS+, DPPH, and •OH radicals. As shown in Figure 6A, the BS of SL-4, SL-5, and SL-7 exhibited ABTS+ clearance rates of 44.72 ± 1.85%, 47.25 ± 1.77%, and 48.59 ± 1.72%, respectively, with no significant differences among strains (*p* < 0.05). The FS of SL-4 and SL-7 showed ABTS+ clearance rates of 93.89 ± 0.21% and 93.94 ± 0.22%, respectively, significantly higher than that of SL-5 (92.57 ± 0.28%) (*p* < 0.05). As shown in Figure 6B, the BS of all three strains displayed weak DPPH scavenging activity (below 10%), while their FS showed markedly enhanced effects. Specifically, SL-5 and SL-7 demonstrated DPPH scavenging rates of 59.28 ± 3.13% and 57.05 ± 4.60%, respectively, significantly more effective than SL-4 at 32.37 ± 3.32% (*p* < 0.05). As shown in Figure 6C, the •OH scavenging rates of BS were 17.71 ± 0.56% for SL-4, 21.22 ± 0.43% for SL-5, and 30.11 ± 0.45% for SL-7, with SL-7 being significantly higher than the other two strains (*p* < 0.05). The FS of all strains exhibited nearly complete •OH scavenging, ranging from 98.36% to 99.15%, with no significant differences (*p* > 0.05).

### 3.5. Identification of Candidate Bacterial Strains

**Figure 7:**
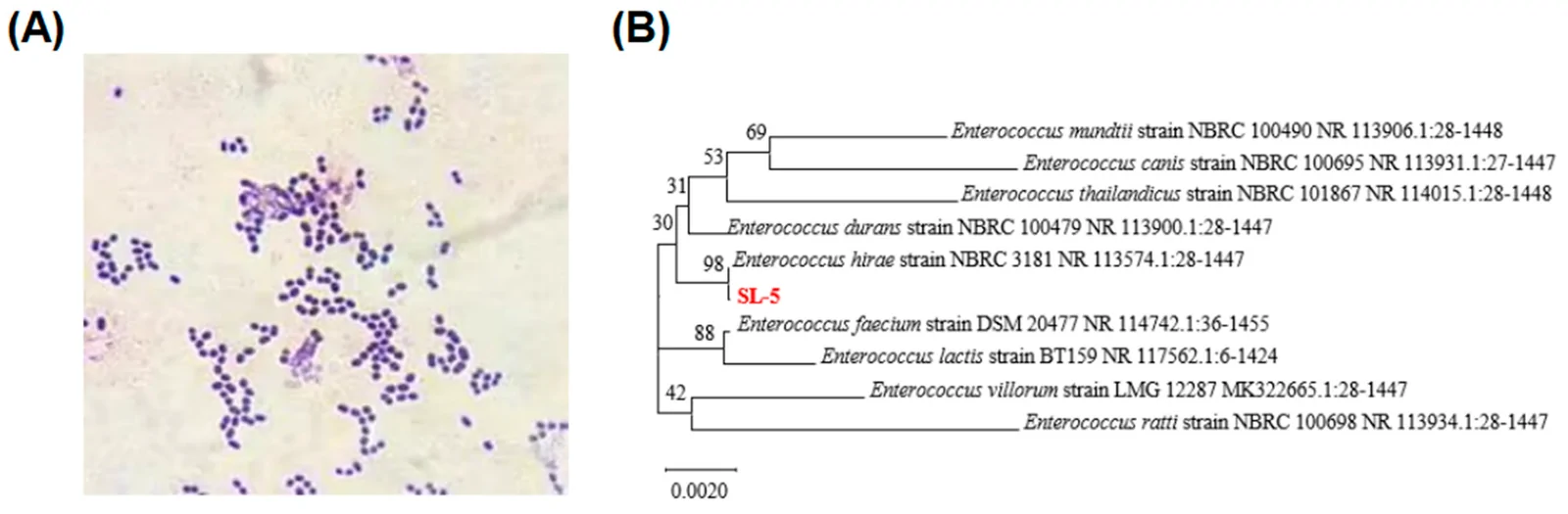
(**A**) Results of Gram stain; (**B**) Phylogenetic tree of candidate strain SL-5.

Based on the comprehensive in vitro and in vivo results, SL-5 was selected for further identification. Microscopic examination revealed purple-colored, spherical cells without spores, consistent with Gram-positive morphology (Figure 7A). BLAST analysis of the 16S rDNA sequence identified the strain as *Enterococcus hirae* (Figure 7B). The strain was deposited in the China Center for Type Culture Collection under accession number CCTCC NO: M20242284.

## 4. Discussion

In this study, a comprehensive screening strategy integrating in vitro functional assays with in vivo validation in a *C. elegans* HUA model was established to identify LAB with significant UA-lowering potential. Among the tested strains, SL-5 exhibited superior performance across multiple evaluation criteria, including UA degradation, purine metabolism, and XOD inhibition. Mechanistically, the functional superiority of SL-5 appears to arise from its multi-target mode of action. On one hand, SL-5 efficiently degrades UA and its purine precursors, particularly adenosine, guanosine, and hypoxanthine, thereby reducing the substrate pool available for XOD-mediated UA synthesis. On the other hand, it directly inhibits XOD activity, suppressing the terminal step of UA formation. This dual action on both precursor supply and terminal synthesis may account for its pronounced UA-lowering effect in vivo, which exceeded that of the other isolates. In addition, its probiotic and antioxidant properties may further contribute by alleviating UA-associated oxidative stress and supporting its persistence and metabolic activity within the intestinal environment.

The UA degradation efficiencies of the selected strains, particularly SL-4, SL-5, and SL-7, were markedly higher than those reported for other LAB strains. For instance, *L. plantarum* JL-3, isolated from Jiangshui, degraded only 40.9% of UA within 24 h [6], while *L. paracasei* JS-3 showed a degradation rate of merely 16.11% over 72 h [18]. The superior degradation efficiency observed in our study may be attributed to differences in initial UA concentration, strain-specific metabolic capabilities, culture conditions, and methodological variations in UA degradation assessment.

The purine-degrading capabilities of SL-4, SL-5, and SL-7, particularly the remarkable efficiency of SL-5 in degrading hypoxanthine (99.29%), highlight their potential for developing functional probiotic solutions. In contrast, previously reported strains such as *L. fermentum* 9–4 demonstrated lower degradation rates for inosine (2.13 mg/L·min) and guanosine (2.78 mg/L·min) [5], and *L. plantarum* L123 displayed minimal activity against hypoxanthine (0.29 mg/L·min) and xanthine (0.16 mg/L·min) [24]. A more comprehensive comparison of the purine-degrading capacities of previously reported LAB strains is summarized in Table 2.

The XOD-inhibitory activity of SL-5 (71.50%) was significantly higher than that of other reported LAB strains, such as *L. fermentum* NCU003018 (30.77%) [10], *W. cibaria* X1 (57.77%) [4], and *L. reuteri* NCUF203.1 (40.46%) [25]. This superior inhibitory effect underscores the potential of SL-5 as a highly effective probiotic strain for suppressing UA synthesis via XOD inhibition. Notably, the inhibitory activity varied across different bacterial fractions, with live bacterial suspensions consistently exhibiting higher activity than heat-inactivated counterparts. Zielińska et al. [26] reported the inhibitory effects of lactic acid bacteria were significantly reduced after heat inactivation, suggesting that bacterial viability and metabolically active processes contribute to their functional activity. Furthermore, it has been shown that both cell-associated components and extracellular metabolites may jointly contribute to biological activity, rather than being attributed to a single fraction [27]. It should be noted that the present study was designed as a screening and functional validation study, and the specific bioactive components and molecular mechanisms underlying XOD inhibition remain to be elucidated. The fraction-based inhibition data suggest that both extracellular metabolites and cell-associated components contribute jointly to XOD inhibition, with heat-stable, low-molecular-weight compounds likely involved. Future studies incorporating metabolite profiling, transcriptomic analysis, and enzyme kinetics are warranted to identify the active compounds and clarify their mechanisms of action, and to distinguish specific inhibitory mechanisms from non-specific chemical interactions.

In the in vivo validation using a hyperuricemic *C. elegans* model, SL-5 reduced UA levels by 52.50%, outperforming other candidate strains and even exceeding the effect of *Weizmannia coagulans* L-BC99, which reduced UA to 322.67 μM in a comparable model [21]. The strong hypouricemic effect of SL-5 may be attributed to its multi-mechanistic action, including direct degradation of UA and its precursors, inhibition of XOD activity, and potential synergistic effects arising from its probiotic and antioxidant properties.

In addition to their core function of lowering UA levels, SL-5 and the other candidate strains in the group also exhibit excellent physiological properties. The strong acidogenesis and high growth rates exhibited by SL-4, SL-5, and SL-7 indicate their potential as starter cultures in food fermentation applications. The self-aggregation ability observed in these strains contributes to intestinal colonization and biofilm formation. Strains with high self-aggregation ability can prevent intestinal tract infections by forming natural barriers on the surface of intestinal cells [28]. The self-aggregation rates of all three strains exceeding 80% after 6 h were notably higher than those reported for *L. fermentum* BGI-AF16 (47.19%) [29] and *W. cibaria* X1 (47.25%) [4]. According to the classification standard established by Montoro et al. [30], where self-aggregation ability is divided into three grades: low (16–35%), medium (35–50%) and high (>50%) [30], our results demonstrate that SL-4, SL-5, and SL-7 strains possess relatively high self-aggregation capabilities.

The tolerance to salinity and nitrite represents an important indicator of fermentation potential [23]. SL-5 demonstrated superior salt tolerance with significantly enhanced growth under 6% NaCl, while all three strains showed strong tolerance to nitrite at 150 mg/L NaNO_2_. The variation in acid tolerance among strains, with SL-4 showing the highest survival rate at pH 3.0, suggests strain-specific adaptations to gastric stress. The exceptional bile salt tolerance observed, particularly in SL-4 (near 100% survival) and SL-5 (88.37%), may be attributed to the presence of bile salt hydrolases, which contribute to the hydrolysis of conjugated bile salts to attenuate toxins on cells [23]. These results compare favorably with previous reports where *L. plantarum* MC14 survived about 70% [9] and *L. fermentum* NCUH003018 survived about 78.9% [10] in 0.3% bile salt concentration.

Furthermore, these strains displayed potent antioxidant capacity. The observed antioxidant capacity primarily originates from metabolites released during fermentation. The FS contains various bioactive compounds secreted by the bacteria, including exopolysaccharides, peptides, organic acids, and other secondary metabolites. These components confer direct free radical scavenging activity, explaining why FS exhibits significantly stronger antioxidant capacity than BS. When compared with previously reported LAB strains, the antioxidant capacity of the SL strains is notably superior. For instance, Cong et al. [31] reported that the FS of *L. rhamnosus* ZYN-0417 and *L. plantarum* ZYN-0221 showed ABTS and •OH scavenging rates of 76.03%, 57.30%, and 66.17%, 46.93%, respectively. Jeong et al. [32] found that the FS of *L. reuteri* MG5149 exhibited ABTS and DPPH scavenging rates of 57.1% and 30.8%, respectively. It should be noted that the antioxidant assays employed in this study (ABTS^+^, DPPH•, and •OH) provide a broad assessment of free radical scavenging capacity but do not specifically target superoxide anions (O_2_•^−^), which are a major reactive oxygen species (ROS) generated during xanthine oxidation by XOD. Given that HUA is accompanied by elevated oxidative stress, and that O_2_•^−^ plays a critical role in exacerbating inflammatory damage, endothelial dysfunction, and gut microbiota dysbiosis, the antioxidant capacity of the strains is functionally relevant to mitigating UA-related metabolic stress. Although the present assays do not specifically capture superoxide anions, the FS of all three strains exhibited nearly complete •OH scavenging activity (98.36–99.15%), and SL-5 and SL-7 displayed considerable DPPH scavenging activity (59.28% and 57.05%, respectively), indicating broad-spectrum and potent free radical scavenging potential. Future studies will incorporate superoxide-specific detection systems, such as the pyrogallol autoxidation assay or WST-1 reduction assay, to directly quantify O_2_•^−^ scavenging activity and further clarify the protective role of these strains against HUA-associated oxidative stress.

The physiological characterization of SL-5 revealed robust growth and stress tolerance, high self-aggregation capacity, and potent antioxidant activity. The identification of *E. hirae* SL-5 adds a novel and promising strain to the limited repertoire of UA-reducing LAB, which has been predominantly focused on *L. plantarum* [6,33], *L. reuteri* [7,19], *L. fermentum* [10,29,34], and *L. gasseri* [12,35], among other strains. Given SL-5 superior UA-degrading, purine-metabolizing, and XOD-inhibitory activities, SL-5 could serve as a valuable host for mining genes encoding UA-degrading enzymes, such as purine nucleoside phosphorylase (PNP) and urate dehydrogenase (Udh). Through whole-genome sequencing, functional gene cloning, and heterologous expression in food-grade safe strains (e.g., *Lactococcus lactis*), the UA-lowering enzyme genes derived from SL-5 hold promise for developing enzyme-based or engineered strain interventions for hyperuricemia, thereby circumventing the safety risks associated with the original strain. 

Importantly, beyond its functional performance in vitro and in vivo models, the isolation of SL-5 from the gut of *T. scripta elegans* suggests that such bacteria may represent a component of the host-associated microbial community contributing to UA metabolism. Given that *T. scripta elegans* are capable of maintaining metabolic homeostasis under relatively high UA levels, the presence of LAB with UA-related metabolic activities raises the possibility that gut microbiota may participate in modulating purine metabolism in this species. Although direct evidence linking specific microbial functions to host UA regulation in reptiles remains limited, our findings provide initial support for a microbe-associated perspective in understanding UA homeostasis in *T. scripta elegans*.

In summary, this study established an integrated screening strategy combining multi-indicator in vitro screening and *C. elegans* model in vivo validation to identify the functionally excellent strain *Enterococcus hirae* SL-5 from the gut of *T. scripta elegans*. Although SL-5 exhibits outstanding performance in UA degradation, purine metabolism, XOD inhibition, and antioxidant activity, due to the opportunistic pathogenicity of its species, it is not recommended for direct application as a probiotic. Instead, SL-5 should be regarded as a valuable genetic resource for mining UA-degrading enzyme genes (e.g., PNP, Udh), providing a theoretical foundation and strain reservoir for the future development of safe and effective enzyme-based or engineered strain interventions for hyperuricemia. This study also provides preliminary experimental evidence for the potential role of reptilian gut microbiota in uric acid metabolism.

## 5. Conclusions

In this study, a comprehensive screening strategy was established to identify LAB strains with anti-hyperuricemia activity. *Enterococcus hirae* SL-5 degraded UA by 76.19% and adenosine, guanosine, and hypoxanthine by 96.62%, 83.50%, and 99.29%, respectively. It also inhibited XOD activity by 71.51% and reduced UA levels in *C. elegans* by 52.50%. *Enterococcus hirae* SL-5 can serve as a valuable genetic resource for the mining of UA-degrading enzyme genes. This study provides an effective screening framework and a novel strain resource for the development of enzyme-based hyperuricemia interventions.

## Figures and Tables

**Table 1:** Degradation rates of purine by different strains.

Purine Degradation Rate	SL-4	SL-5	SL-7	SL-10	SL-17
Adenosine (%)	97.07 ± 2.93 a	96.62 ± 0.48 a	96.03 ± 0.23 a	93.71 ± 0.23 a	94.73 ± 0.08 a
Adenosine mg/(L*min)	11.80 ± 0.41 c	11.63 ± 0.06 c	10.56 ± 0.03 b	9.35 ± 0.04 a	9.52 ± 0.01 a
Guanosine (%)	74.13 ± 3.67 b	83.50 ± 1.54 c	82.70 ± 2.35 c	30.04 ± 0.86 a	30.89 ± 0.86 a
Guanosine mg/(L*min)	11.51 ± 0.26 b	12.49 ± 0.33 c	12.36 ± 0.18 c	5.17 ± 0.05 a	5.41 ± 0.12 a
Inosine (%)	38.22 ± 0.29 bc	36.60 ± 0.09 b	39.67 ± 1.62 c	26.71 ± 0.50 a	25.95 ± 0.32 a
Inosine mg/(L*min)	2.17 ± 0.64 b	2.02 ± 0.60 b	2.28 ± 0.39 b	1.63 ± 0.05 a	1.33 ± 0.03 a
Adenine (%)	36.05 ± 5.56 bc	27.83 ± 3.66 ab	42.69 ± 5.77 c	18.57 ± 0.84 a	27.68 ± 0.55 ab
Adenine mg/(L*min)	3.63 ± 0.66 c	2.55 ± 0.64 bc	3.69 ± 0.51 c	2.07 ± 0.10 a	2.25 ± 0.00 ab
Xanthine (%)	13.39 ± 2.50 b	14.79 ± 2.66 b	15.89 ± 4.88 b	8.77 ± 3.49 ab	3.90 ± 2.16 a
Xanthine mg/(L*min)	0.47 ± 0.05 ab	0.49 ± 0.05 c	0.56 ± 0.53 c	0.32 ± 0.08 ab	0.24 ± 0.11 a
Hypoxanthine (%)	96.04 ± 2.53 b	99.29 ± 0.56 b	97.14 ± 3.82 b	54.53 ± 4.53 a	52.51 ± 1.97 a
Hypoxanthine mg/(L*min)	30.83 ± 0.34 b	32.05 ± 1.53 b	31.89 ± 0.52 b	20.69 ± 0.62 a	20.32 ± 0.35 a

Notes: Different lowercase letters within each row indicate statistically significant differences among strains (*p* < 0.05). * denotes multiplication.

**Table 2:** Degradation rates of purines by reported LAB.

Strains	Guanosine Degradation Rate	Inosine Degradation Rate	Adenine Degradation Rate	Xanthine Degradation Rate	Hypoxanthine Degradation Rate	References
*L. plantarum* MC14	63.87%	74.76%	/	/	/	[9]
*L. fermentum* 9-4	58.51%	47.74%	/	/	/	[5]
*L. casei* NCUH011026	80.98%	73.96%	/	/	/	[10]
*L. fermentum* NCU003012	72.21%	67.59%	/	/	/	
*Weissella cibaria* X1	45.26%	99.23%	/	/	/	[4]
*L. brevis* DM9218	99.64%	99.31%	/	/	/	[8]
*L*. *gasseri* PA-3	100.00%	100.00%	/	/	/	[12]
*L. plantarum* L123	96.88%	100.00%	/	11.23%	14.27%	[24]

Note: / indicates that the corresponding purine degradation experiments were not performed.

## Data Availability

The original data presented in the study are openly available in Supplementary Material. Further inquiries can be directed to the corresponding author.

## Supplementary Materials

The following supporting information can be downloaded at: https://www.mdpi.com/article/10.3390/microorganisms14102336/s1.

## Abbreviations

The following abbreviations are used in this manuscript:

HUA	Hyperuricemia
LAB	lactic acid bacteria
XOD	xanthine oxidase
UA	uric acid
*C. elegans*	*Caenorhabditis elegans*
HPLC	high-performance liquid chromatography
NGM	nematode growth medium
SM	S-medium
CON	control
MOD	model
PBS	phosphate-buffered saline
